# Optical Filter-Embedded Fiber-Optic Radiation Sensor for Ultra-High Dose Rate Electron Beam Dosimetry

**DOI:** 10.3390/s21175840

**Published:** 2021-08-30

**Authors:** Dong-Hyeok Jeong, Manwoo Lee, Heuijin Lim, Sang-Koo Kang, Kyohyun Lee, Sang-Jin Lee, Hyun Kim, Woo-Kyung Han, Tae-Woo Kang, Kyoung-Won Jang

**Affiliations:** Research Center, Dongnam Institute of Radiological and Medical Sciences, Busan 46033, Korea; physics@dirams.re.kr (D.-H.J.); mwlee@dirams.re.kr (M.L.); heuijin@dirams.re.kr (H.L.); radiation@dirams.re.kr (S.-K.K.); kyohyun11@dirams.re.kr (K.L.); jak7513@dirams.re.kr (S.-J.L.); kh2532@dirams.re.kr (H.K.); wkhan@dirams.re.kr (W.-K.H.); xodn5492@dirams.re.kr (T.-W.K.)

**Keywords:** fiber-optic radiation sensor, ultra-high dose rate electron beam, FLASH radiotherapy

## Abstract

FLASH radiotherapy is an emerging radiotherapy technique used to spare normal tissues. It employs ultra-high dose rate radiation beams over 40 Gy/s, which is significantly higher than those of conventional radiotherapy. In this study, a fiber-optic radiation sensor (FORS) was fabricated using a plastic scintillator, an optical filter, and a plastic optical fiber to measure the ultra-high dose rate electron beams over 40 Gy/s used in FLASH radiotherapy. The radiation-induced emissions, such as Cherenkov radiation and fluorescence generated in a transmitting optical fiber, were spectrally discriminated from the light outputs of the FORS. To evaluate the linearity and dose rate dependence of the FORS, the outputs of the fiber-optic radiation sensor were measured according to distances from an electron scattering device, and the results were compared with those of an ionization chamber and radiochromic films. Finally, the percentage depth doses were obtained using the FORS as a function of depth in a water phantom. This study found that ultra-high dose rate electron beams over 40 Gy/s could be measured in real time using a FORS.

## 1. Introduction

Radiation therapy techniques such as intensity-modulated radiation therapy (IMRT) and image-guided radiation therapy (IGRT) have been developed to eradicate tumors with minimal damage to normal tissues [1,2]. FLASH radiotherapy (FLASH-RT) is an emerging radiotherapy technique used to spare normal tissues [3,4,5,6]. It employs ultra-high dose rate radiation beams over 40 Gy/s, which is significantly higher than those of conventional radiotherapy; for example, the dose rate of conventional radiation therapy using clinical linear accelerators (LINACs) is approximately 0.03 Gy/s [4]. FLASH-RT ultra-high dose rate radiation beams enhance the differential effect between the tumors and normal tissues and destroy tumors while sparing normal tissues from radiation damage [6]. However, to date, the biological mechanism of FLASH-RT remains undefined [7], and only a few facilities can conduct FLASH-RT research using electron and proton beams because of the difficulty of achieving an ultra-high dose rate of over 40 Gy/s. From the perspective of radiation dosimetry, real-time measurement of ultra-high dose rate radiation beams is challenging. Conventional ionization chambers (ICs), widely used in radiotherapy dosimetries, have a dose-per-pulse (DPP) dependence that is induced by the ion recombination effect and the outputs of ICs require a correction process for DPPs greater than approximately 0.01 Gy/pulse [8]. Radiochromic films can also be used in FLASH-RT dosimetry; however, dosimeters of this type are intrinsically incapable of real-time measurements [9].

Over the last several decades, optical fiber-based physical sensors have been developed and used in various nuclear and medical facilities [10,11,12]. In particular, fiber-optic radiation sensors (FORSs) have desirable characteristics for therapeutic radiation dosimetry [13]. When FORSs are exposed to ionizing radiation, radiation-induced light signals are emitted from the sensing parts, which are scintillators, or the optical fibers themselves used to transmit light to photodetectors. Light signals travelling via optical fibers are rarely affected by ambient conditions such as temperature, pressure, humidity, and electromagnetic fields [14]. In addition, water-equivalent components of FORSs, such as polymethyl methacrylate (PMMA) and polystyrene (PS), allow the measurement of radiation doses without complicated correction processes. Furthermore, the outputs of FORSs have been reported to have dose rate and energy independence for therapeutic radiation beams [15].

In this study, a FORS was fabricated using a plastic scintillator, an optical filter, and a plastic optical fiber (POF) for dosimetry of ultra-high dose rate electron beams and was subsequently evaluated with electron beams from a 6 MeV LINAC at Dongnam Institute of Radiological and Medical Sciences (DIRAMS), developed as a compact LINAC system that recently implemented ultra-high dose rate electron beams for preclinical FLASH-RT studies [16]. The radiation-induced emissions (RIEs), such as Cherenkov radiation and fluorescence generated in a transmitting optical fiber, were spectrally discriminated from the light outputs of the FORS by using an optical filter. To evaluate the linearity and dose rate dependence of the FORS, the outputs of the FORS were measured according to the distances from the electron scattering device, and the results were compared with those of an IC and radiochromic films. In addition, the percentage depth doses (PDDs) were measured as a function of depth in the water phantom. Finally, through the results, the FORS-based dosimetry technique for real-time FLASH beam measurement was evaluated.

## 2. Materials and Methods

A cylindrical plastic scintillator (BCF-60, Saint-Gobain Crystals, Hiram, OH, USA), having a diameter and length of 1.0 mm and 10 mm, respectively, was used. Scintillators of this type have a core/cladding structure. The primary materials of the core and cladding are PS and PMMA, respectively. Since the number of electrons per gram of PS and PMMA is $3.24\times 10^{23}$, which is very close to that of water ($3.34\times 10^{23}$), they are regarded as water-equivalent materials in radiotherapy dosimetry [17]. The refractive indices of PS and PMMA are 1.60 and 1.49, respectively, and the numerical aperture (NA), which denotes the light-gathering power, is 0.58. Although several BCF series scintillators such as BCF-10, BCF-12, and BCF-20 have higher scintillation outputs and faster decay times than those of the BCF-60 scintillator, BCF-60, including 3-hydroxyflavone, has radiation resistance which is favorable for measuring high-dose radiation. The peak emission wavelength of the BCF-60 scintillator was 530 nm, and the number of photons per MeV and decay time were approximately 7100 and 7.0 ns, respectively.

A multimode step-index POF (GH-4001, Mitsubishi Chemical, Tokyo, Japan) was used to transmit the BCF-60 scintillation to a photodetector. The POF consists of PMMA as the core and a fluorinated polymer for the cladding. The refractive indices of the core and cladding were 1.492 and 1.402, respectively, and the NA was 0.50. Further, the POF has a maximum transmission loss of 0.19 dB/m at 650 nm collimated light. The diameter and length of the POF used were 1.0 mm and 20 m, respectively.

**Figure 1:**
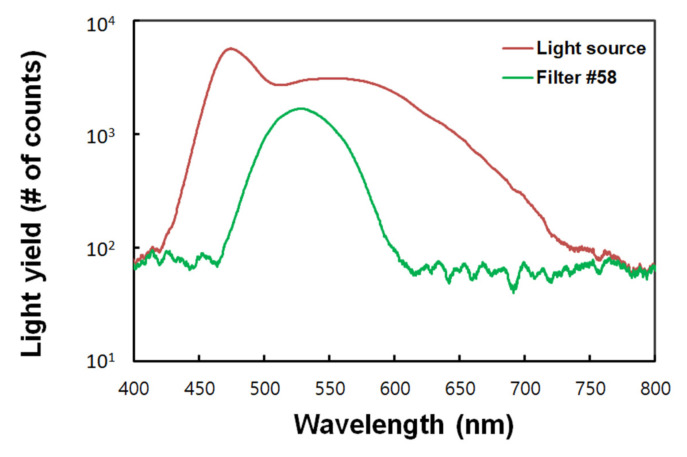
Light transmittance characteristics of Kodak Wratten filter No. 58.

A Wratten gelatin filter No. 58 (Kodak, Rochester, NY, USA) is embedded in the FORS for discriminating RIEs generated in the transmitting POF. Filters of this type transmit light of 470–600 nm wavelengths and has a maximum transmittance at approximately 530 nm, which is similar to the peak emission wavelength of BCF-60. The light transmittance characteristics of the filter are shown in Figure 1, which were obtained with and without the filter using a white light-emitting diode and a spectrometer.

To measure the light outputs of the FORS, a spectrometer (Maya 2000 Pro, Ocean Insight, Orlando, FL, USA) was used as a photodetector. The spectral range of the spectrometer was 200–1100 nm. The peak quantum efficiency and the signal-to-noise ratio of the spectrometer were 75% and 450:1, respectively. In addition, the range of integration time of the spectrometer was 7.2 ms–5.0 s. The integration time and the data acquisition time were set to 100 ms and 3.0 s, respectively; since the irradiation time of the electron beams was within 1.0 s throughout this research, the acquisition time was set to 3.0 s in total, including the 1.0 s interval before and after the irradiation time.

**Figure 2:**
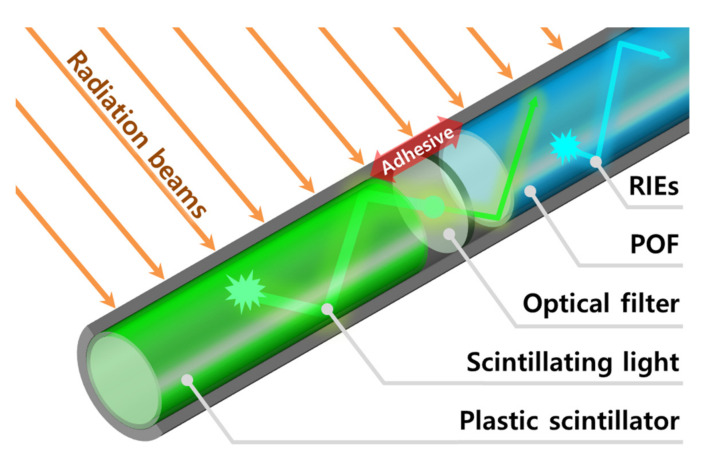
Structure of an optical filter-embedded FORS.

Figure 2 shows the structure of an optical filter-embedded FORS. As shown in the figure, the FORS used in this study consisted of a plastic scintillator, optical filter, and POF. When the radiation beams are directed onto the FORS, the scintillation generated by the scintillator is filtered by an optical filter and transmitted through the POF to the spectrometer. However, RIEs, such as fluorescence and Cherenkov radiation produced in the POF, are also transmitted to the spectrometer. In general, the wavelength range of RIEs covers that of the scintillation generated by a scintillator, and the intensity of RIEs generated in optical fibers for transmission varies with the irradiated length of optical fibers. Although the RIEs themselves can be significant signals in radiation detection, the ones generated in the optical fibers for transmission are regarded as noise signals. Typically, they can be removed by the subtraction method using an additional background optical fiber [18]. However, this method uses a two-channel photodetector and requires a calibration process for each channel. In our work, the RIEs were removed using a wavelength discrimination method using an optical filter [19]—the optical filter limits the spectrum of the scintillation generated by the scintillator, allowing the RIEs of the POF to be spectrally distinguishable.

**Figure 3:**
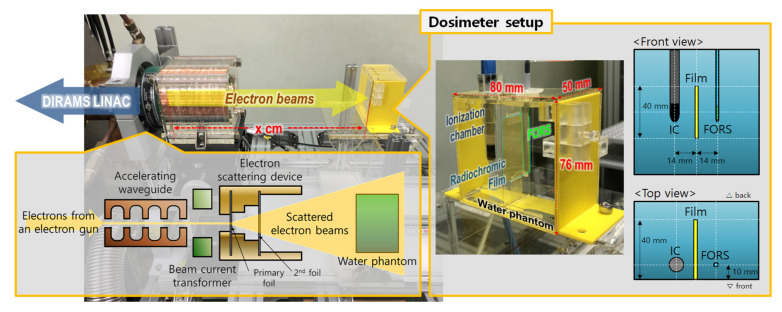
Experimental setup for measuring ultra-high dose rate electron beams.

The experimental setup for measuring the ultra-high dose rate electron beams generated by the DIRAMS LINAC using a FORS is shown in Figure 3. The DIRAMS LINAC operates at the radio frequency (RF) of 5.712 GHz and was designed to produce 6 MeV electron beams at the 2.5 MW RF power [20]. The repetition rate, current, and width of the electron beam pulses were 100 Hz, 50 mA, and 2.78 μs, respectively [20]. In the DIRAMS LINAC, electrons emitted from the electron gun are accelerated in the accelerating waveguide, and high-energy electron pencil beams are incident on the scattering device. Finally, the scattered electron beams irradiate the FORS in a small water phantom at several different distances from the primary foil of the electron scattering device.

The small water phantom used in this research is designed to insert three types of dosimeters and is fabricated via 3D printing; in addition to the FORS, an IC (Semiflex Chamber Type 31010, PTW, Freiburg, Baden-Württemberg, Germany) and radiochromic films (Gafchromic MD-V3 film, Ashland, Wilmington, DE, USA) were employed for the comparison. In the comparative experiments, for simultaneous measurement using the three types of dosimeters, the dosimeters were installed with 14 mm horizontal intervals in the water phantom as shown in Figure 3.

As mentioned above, because ICs undergo ion recombination, radiochromic films are used to measure the absorbed doses. The films used in this experiment were calibrated with 6 MeV electron beams emitted from a medical LINAC (Infinity, Elekta, Stockholm, Sweden). The calibration was conducted at the maximum dose depth in the water phantom with a 100 cm source-to-surface distance (SSD), and the irradiated doses on the films were from 100 to 5000 MU at the dose rate of 400 MU/min. Finally, the films were scanned with a flatbed scanner (10000XL, Epson, Suwa-shi, Nagano, Japan) and analyzed using the FilmQApro software (Ashland, Wilmington, DE, USA).

## 3. Experimental Results and Discussions

**Figure 4:**
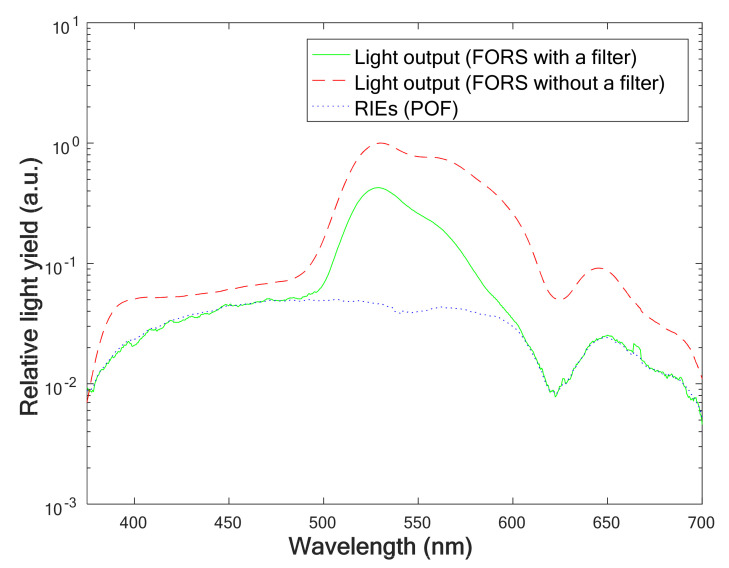
Measured spectra of light outputs of FORSs and the POF.

To measure only the scintillation signal generated in the scintillator, RIEs such as Cherenkov radiation and fluorescence generated in the POF must be removed. In this study, the RIEs generated by the POF were removed using the wavelength discrimination method with an optical filter placed between the scintillator and the light transmitting optical fiber as shown in Figure 2. Figure 4 shows the measured spectra of the light outputs of the FORSs (with and without the optical filter) and the POF. Although the light output spectrum of the BCF-60 scintillator has the peak at the wavelength of 530 nm, the wide wavelength range makes it difficult to distinguish the RIEs in the light output of FORSs. To spectrally discriminate the RIEs, an optical filter was embedded between the scintillator and the POF. The optical filter limits the scintillation wavelengths of the scintillator except for specific wavelengths; therefore, the RIEs of a POF can be easily removed from the light output of a FORS. The scintillation spectrum generated by the BCF-60 scintillator was falsified by the filter. Since the filter was used, the peak intensity of the light output generated by the FORS was reduced by approximately 57%. Although light reduction may affect sensitivity of a FORS, it does not significantly affect the results because relatively high-dose electron beams are used in preclinical FLASH studies.

**Figure 5:**
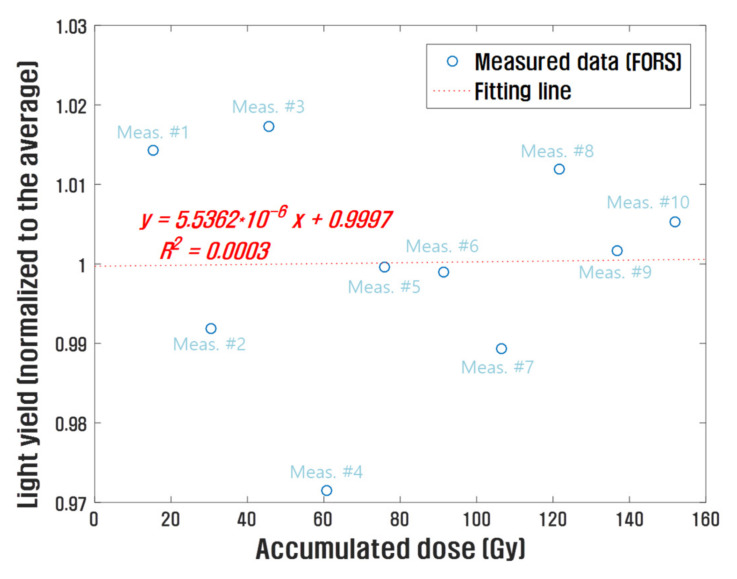
Fluctuation of the measured scintillation generated in the FORS.

Figure 5 shows the fluctuation in the measured scintillations generated in the FORS. In this experiment, the FORS was irradiated with 10 electron beam pulses for each measurement, and the DPP of the beams was approximately 1.52 Gy/pulse. The measurements were conducted 10 times at 10 mm depth of the water phantom. The scintillation outputs varied within ±3%, and the relative standard deviation between the measured scintillation outputs was approximately 1.36%. This fluctuation result can also be expressed as degradation of scintillation according to the electron beam irradiation. For each measurement, the FORSs were exposed to a total dose of approximately 15.2 Gy. Typically, ionizing radiation breaks polymer chains of plastic materials and creates radicals that absorb ultraviolet (UV) light [21]. The peak emission wavelength of BCF-60 used in this study was 530 nm, which is significantly different from the UV absorption wavelength of a polymer material upon irradiation with ionizing radiation. In addition, the optical filter embedded in the FORS can minimize the UV signals incident on the POF. In our experiment, the attenuation tendency of the scintillation caused by the irradiation of ultra-high dose rate electron beams was negligible. Further, the gradient of the fitting line for the measured data was very small—approximately $5.5\times 10^{-5}$—and R-squared was 0.0003.

**Figure 6:**
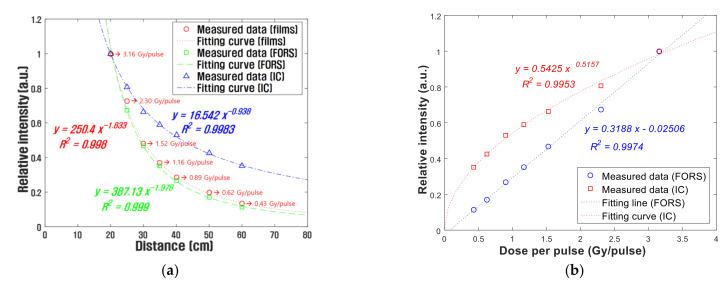
(**a**) Relative outputs of the films, IC, and FORS according to distances from the electron scattering device; (**b**) relative outputs of the FORS and IC according to DPP, as measured using a radiochromic film.

To evaluate the dose rate dependence of the FORS in ultra-high dose rate conditions over 40 Gy/s, the relative outputs of the films, IC, and FORS according to distances from the electron scattering device were obtained with a dosimeter setup shown in Figure 3. The results are shown in Figure 6. In this experiment, the films were used as a reference dosimeter to measure the absorbed doses in the water phantom. As in the previous experiment, the dosimeters were irradiated to ten electron beam pulses at each distance. As a result, the outputs of the films, IC, and FORS decreased with the inverse power laws as the distance from the scattering device increased. The decreasing trends of the films and the FORS were very similar, but the decreasing trend of ICs was significantly different from those of the films and FORS. This phenomenon is caused by the ion recombination effect of the IC, which leads to the outputs from the IC in the high-dose rate region, at short distances, being relatively small. Therefore, the decreasing gradient of the outputs from the IC according to the distance was smaller than those of the FORS and the films. The DPPs obtained using the films varied from 0.43 Gy/pulse to 3.16 Gy/pulse.

The relationship between the DPPs obtained using the films and the relative outputs of the FORS and IC are shown in Figure 6b. The ion recombination effect in the IC can be seen more clearly. As the DPP increased, the output of the FORS increased linearly, whereas the output of IC increased in the power form. At the 100 Hz repetition rate, the minimum dose rate was 43 Gy/s at the distance of 60 cm, which was greater than the maximum dose rate (12 Gy/s) for 99% saturation ion collection efficiency of the IC used in this study [22]. The voltage supplied to the IC was +300 V throughout this research; according to the IC specification, the maximum is 500 V. Although higher voltages can improve the IC performance in ultra-high dose rate conditions, the correction process is required for accurate measurements. In contrast, in the case of a FORS, real-time dosimetry in ultra-high dose rate conditions is possible without a complicated correction process.

**Figure 7:**
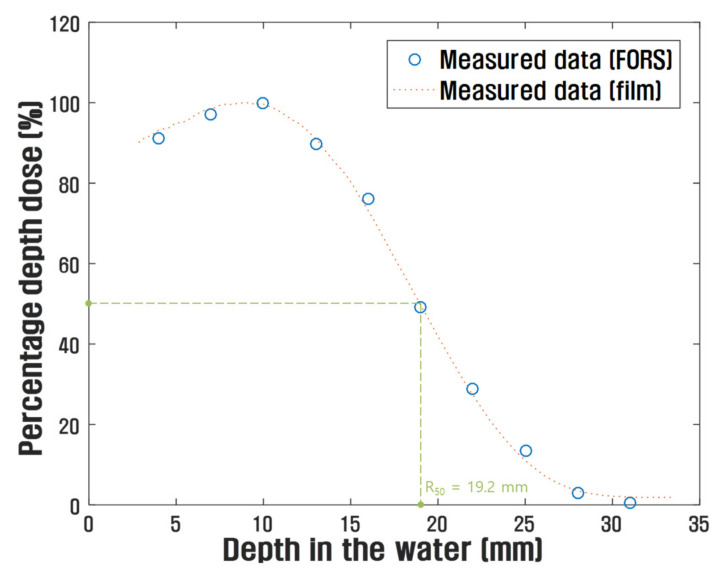
Measured PDDs using the FORS and the films.

Figure 7 shows the measured PDDs of ultra-high dose rate electron beams using the FORS. In this study, 10 electron beam pulses were irradiated on the FORS at each water phantom depth. In addition, the PDD curve of the film was used for comparison. In our measurement, the PDDs obtained using the FORS and the film were very similar, and the mean difference between the PDDs using the FORS and the film was approximately 1.3%. Moreover, the measured maximum dose depths were approximately 10 mm, and the PDDs decreased sharply after reaching the maximum dose depth. Typically, PDD curves for electron beams drop rapidly, and this characteristic offers a distinct clinical advantage over conventional X-ray beams. The drop-off depth in the water phantom is related to the electron beam energy, which can be estimated using PDD curves. Using R_50_, which is the depth at half-maximum on the PDD curve, as an energy index, the mean energy ($\bar{E_{0}}$) of electron beams can be calculated as follows [17]:

(1) $$\bar{E_{0}}\left(\mathrm{MeV}\right)=2.33\times R_{50}.$$

In this study, the R_50_ obtained using the FORS was 19.2 mm, and the calculated mean energy of the electron beams was approximately 4.5 MeV. Moreover, although a 6 MeV LINAC was used to generate electron beams, the lower-energy electron beams were exploited throughout this research owing to the adjustment of the LINAC conditions for increasing the dose rate [23].

## 4. Conclusions

FORSs have several advantages in the field of radiotherapy dosimetry, including water equivalence, high spatial resolution, dose rate independence, and robustness against ambient conditions. In particular, the dose-rate independence of FORSs is very advantageous in the ultra-high dose rate radiation dosimetry of FLASH-RT. In this study, an optical filter-embedded FORS was fabricated for the dosimetry of ultra-high dose rate electron beams generated using the DIRAMS LINAC. The RIEs produced in the FORS were spectrally discriminated from the total light output of the FORS. The FORS outputs varied within the range of ±3% in repeated measurements, the relative standard deviation was approximately 1.36%, and radiation-induced attenuation of the light outputs of the FORS was negligible; here, since the fluctuation result also includes the variation of the beam output, the actual fluctuation of the FORS output is considered to be smaller. Further, the outputs of the FORS were consistent with those of the films. In addition, the PDDs obtained with the FORS are consistent with the film results. Thus, through this study, it was found that ultra-high dose rate electron beams over 40 Gy/s could be measured in real time using a FORS. Further work will involve utilizing Cherenkov radiation generated from POFs by ultra-high dose rate electron beams for dosimetry. FORSs are expected to be effective in ultra-high dose rate radiation dosimetry of FLASH-RT.
